# Analysis of the effect of daily stress on the skin and search for genetic loci involved in the perceived stress of an individual

**DOI:** 10.1002/ski2.110

**Published:** 2022-04-01

**Authors:** Yu Inoue, Yuichi Hasebe, Toshio Igarashi, Mika Kawagishi‐Hotta, Ryosuke Okuno, Takaaki Yamada, Seiji Hasegawa

**Affiliations:** ^1^ Research Laboratories Nippon Menard Cosmetic Co., Ltd Nagoya Aichi Japan; ^2^ Nagoya University‐MENARD Collaborative Research Chair Nagoya University Graduate School of Medicine Nagoya Aichi Japan

## Abstract

**Background:**

Stress may have various effects on our bodies. In particular, the skin may be readily influenced by stress. In addition, there are individual differences in the way we feel stress, suggesting the involvement of genetic factors in such individual differences.

**Objectives:**

In this study, we analysed the influence of stress on skin condition and ageing involving Japanese females, and investigated single nucleotide polymorphisms (SNPs) that influence perceived stress of an individual.

**Methods:**

We collected genotype data from 1200 Japanese females. At the same time, a questionnaire was conducted on the degree of stress that each subject feels on a daily basis and the current skin condition. We analysed the effects of stress on skin condition and searched for SNPs related to individual stress susceptibility by genome‐wide association studies.

**Results:**

Our data suggested that stress influences skin condition and ageing, as previously reported. And, we found rs74548608 as a SNP that affects perceived stress of an individual. This SNP is located on the upstream of Patched‐1, which is a gene that functions as a sonic hedgehog receptor.

**Conclusions:**

Our study has identified new genetic factors for perceived stress of an individual in the Japanese female. The SNP found in this study may be a candidate factor important for understanding the perceived stress of an individual of Japanese.

## Graphical abstract

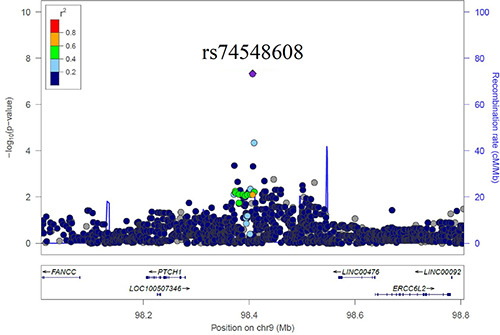

1

**What's already known about this topic?**

- Stress may have various effects on our bodies. In particular, the skin may be readily influenced by stress.
- In addition, there are individual differences in the way we feel stress, and genetic factors are thought to be involved.

**What does this study add?**

- Our data suggested that stress influences skin condition and ageing in Japanese female.
- In this study, we found rs74548608 as an single nucleotide polymorphism (SNP) involved in perceived stress of an individual through a genome‐wide association studies involving Japanese females. To our knowledge, rs74548608 was first reported as an SNP associated with perceived stress in Japanese females.
- This SNP is located on the upstream of Patched‐1, which is a gene that functions as a sonic hedgehog receptor.

## 2 INTRODUCTION

Factors for individuals' stress include environmental factors, such as noises and the weather, physical factors, such as diseases and injuries, mental factors, such as anxiety and tension, and social factors, such as busyness and heavy responsibilities. Stress may have various effects on our bodies. In particular, the skin may be readily influenced by stress. Excessive stress affects the autonomic nerves or hormone balance, inducing various skin troubles, such as the promotion of skin ageing and exacerbation of dermal diseases.^1^, ^2^, ^3^, ^4^, ^5^ Furthermore, it was reported that stress reduced the epidermal barrier function.^6^, ^7^ In addition, there are individual differences in the way we feel stress,^8^, ^9^ and the involvement of genetic factors is suggested.

A genome‐wide association study (GWAS) is a method to statistically detect trait‐associated gene polymorphisms by comprehensively investigating the association between differences in traits among populations and those in DNA sequences over a genome‐wide range. For GWAS, hundreds of thousands to millions of single nucleotide polymorphisms (SNPs) are primarily used as genetic markers. As most SNPs exist in areas other than gene or protein expression‐regulating areas, they do not change genetic characteristics. However, some SNPs may induce genetic individual differences. Recently, various studies regarding stress and genetic factors have been conducted,^10^, ^11^ but most studies involved Europeans and Americans; few studies involving Asians, especially Japanese, have been carried out.

In this study, we analysed the influence of stress on skin condition and ageing involving Japanese females, and investigated SNPs that influence perceived stress of an individual.

## 3 MATERIALS AND METHODS

### 3.1 Study population

The subjects were healthy adults (1200 females living in Japan) from whom written informed consent regarding study participation was obtained. They were similar to those enroled in our previous study.^12^ Prior to this study, its protocol was approved by the Ethics Review Board of our company.

### 3.2 Genotype quality control and imputation

Genome data were collected from the subjects, as previously described.^12^ Briefly, saliva was collected from each subject using a saliva sampling kit (Genesis Healthcare). Genome DNA was extracted using a Maxwell RSC Stabilised Saliva DNA Kit (Promega). In the DNA samples obtained, genotyping was conducted using an Asian Screening Array (ASA) (Illumina). As a result, approximately 650 000 SNPs were detected.

PLINK software is a commonly used whole‐genome association analysis toolset (www.cog‐genomics.org/plink/1.9/).^13^ Using PLINK software for genetic statistical analysis, subjects and SNPs with a call rate of < 0.98 were excluded from the above 1200 subjects and SNPs. Furthermore, SNPs with a *p*‐value of < 1 × 10^−3^ on the Hardy‐Weinberg equilibrium probability test or a minor allele frequency (MAF) of < 5% were excluded. Since the presence of relatives in the subjects affects the results of GWAS analysis, relatives were excluded from the samples using the PI_HAT value, which is an index of the degree of kinship among samples. Specifically, using PLINK, individual pairs with a PI_HAT of ≥0.25 were detected, and individuals with a low individual call rate in respective pairs were excluded from the subjects to be analysed. In addition, clustering with multi‐dimensional scaling was conducted based on the genotype data using PLINK software. In addition to the results, Japanese‐derived individuals were selected based on the questionnaire data on birthplaces. The number of individuals selected as subjects to be analysed through quality control was 1040, and that of SNPs was 299687. Genotype imputation is a procedure to estimate the alleles of ungenotyped SNPs based on the linkage disequilibrium (LD) compared with directly genotyped markers using an appropriate reference population.^14^ In this study, genotype imputation was performed using Eagle software^15^ and Minimac 4 software^16^ based on the 1000 Genomes Project Phase 3 reference panel.^17^ After imputation, SNPs with a call rate of < 0.98, those with a *p*‐value of < 1 × 10^−3^ on the Hardy–Weinberg equilibrium probability test, those with an Rsq (Imputation quality score) of <0.3 as an index of the accuracy of imputation results, and those with an MAF of <5% were excluded. Finally, 6257786 SNPs were estimated.

### 3.3 Questionnaire regarding daily perceived stress and skin condition

Each subject answered a self‐administered questionnaire. To the question: ‘Do you feel stressed in your daily life?”, each subject was instructed to select one of the following options: (1) I don't feel stressed (low), (2) I sometimes feel stressed (middle), and (3) I feel very stressed (high). In addition, to the question: “Please answer your current skin condition”, each subject was instructed to select one of the following options (1) Good, (2) Normal, (3) Somewhat bad, and (4) Bad. Furthermore, each subject was instructed to select an image reflecting her current skin condition from 6‐step images (Figure S1) regarding skin ageing. Regarding the ‘frequency of the use of sunscreen”, each subject was instructed to select an answer from (1) Rarely, (2) Occasionally, and (3) Daily. Also, regarding the ‘smoking habit”, each subject was instructed to select an answer from (1) Never smoked, (2) Previously smoked, (3) Currently smoking 1–10 cigarettes/day, and (4) Currently smoking 11 or more cigarettes/day.

### 3.4 Analysis of the influence of ageing on daily perceived stress

Using the chi square test, daily perceived stress was compared between 2 groups: subjects aged <50 years (*n* = 558, mean age: 40.1 years) and those aged ≥50 years (*n* = 482, mean age: 59.3 years). A *p*‐value of 0.05 was regarded as significant.

### 3.5 Association analysis of stress and skin condition

With respect to the association between stress and skin condition, multiple regression analysis was performed using the grade of skin condition as an objective variable and the degree of stress (1. Don't feel stressed, 2. Sometimes feel stressed, and 3. Feel very stressed) and age as explanatory variables, and a *p*‐value for the regression coefficient of the above degree of stress was evaluated. The frequency of the use of sunscreen and smoking habit were also added as explanatory variables. As 8 traits for skin ageing were investigated, Bonferroni correction was applied. A *p*‐value of 0.00625 was regarded as significant (0.05/8). Furthermore, a *p*‐value of 0.05 was regarded as nominally significant. In this study, the data of 1036 subjects who answered all the questions of the questionnaire were analysed.

### 3.6 Search for SNPs involved in perceived stress of an individual

We used PLINK to perform the genome wide association analysis for the phenotype using multiple linear regression with an additive genetic model. Age was used as covariates. The genome wide significance threshold (5 × 10^−8^) was used to assess statistical significance.

## 4 RESULTS

Past reports have shown that daily perceived stress decreases with ageing after the age of 50 years.^18^, ^19^, ^20^, ^21^ A study in a Japanese population also showed that the types of stress perceived daily change around the age of 50 years.^21^ Specifically, stress related to the subjects' work and education of their children decrease, and stress related to their own diseases increase after the age of 50 years. Initially, we divided the subjects into 2 groups: young (age: <50 years, *n* = 558, mean age: 40.1 years) and elderly (age: ≥50 years, *n* = 482, mean age: 59.3 years) groups, and examined whether or not there are ageing‐related changes in daily perceived stress. As a result, the rate of persons feeling very stressed in daily living in the elderly group was lower than in the young group. On the other hand, the rate of persons who do not feel stressed was higher in the elderly group (Table 1), suggesting that daily perceived stress reduces with ageing. This was consistent with the results of previous studies.^18^, ^19^, ^20^, ^21^ Next, we investigated the influence of stress in daily living on the skin condition and skin ageing. As a result, stress was significantly associated with skin condition (*p* = 0.0000128, *β* = 0.170) and wrinkles between the eyebrows (*p* = 0.00578, *β* = 0.109) (Table 2). It was associated with wrinkles at the corners of the eyes (*p* = 0.0379, *β* = 0.105), nasolabial folds (*p* = 0.0485, *β* = 0.102), and wrinkles on the corners of the mouth (*p* = 0.0496, *β* = 0.0948), showing nominally significant differences (*p* < 0.05) (Table 2). These results were the same after correction for the frequency of the use of sunscreen or smoking habit (Table S1). Therefore, it was suggested that stress makes skin condition worse and promotes skin ageing, as previously indicated.

**TABLE 1:** Comparison of daily perceived stress in the younger group (under 50) and the older group (over 50)

Stress	Groups				χ2	*p*
	Under 50 (*N* = 558)		Over 50 (*N* = 482)			
	*N*	%	*N*	%		
Low	32	5.7	36	7.5	16.6	0.0002
Middle	352	63.1	349	72.4		
High	174	31.2	97	20.1		

**TABLE 2:** Association results of the stress with skin condition and skin ageing

Skin condition and signs of skin ageing	Adjustments	
	Age	
	*β*	*p*
Skin condition	0.170	0.0000128
Wrinkles between the eyebrows	0.109	0.00578
Wrinkles on the forehead	0.046	0.286
Wrinkles on the inner corner of the eye	0.057	0.175
Wrinkles at the corners of the eyes	0.105	0.0379
Nasolabial fold	0.102	0.0485
Wrinkles on the corners of the mouth	0.0948	0.0496
Eyelid sagging	0.038	0.304

*Note*: Association tests were adjusted for age.

Abbreviations: *β*, beta coefficient; *p*, *p*‐value.

**FIGURE 1:**
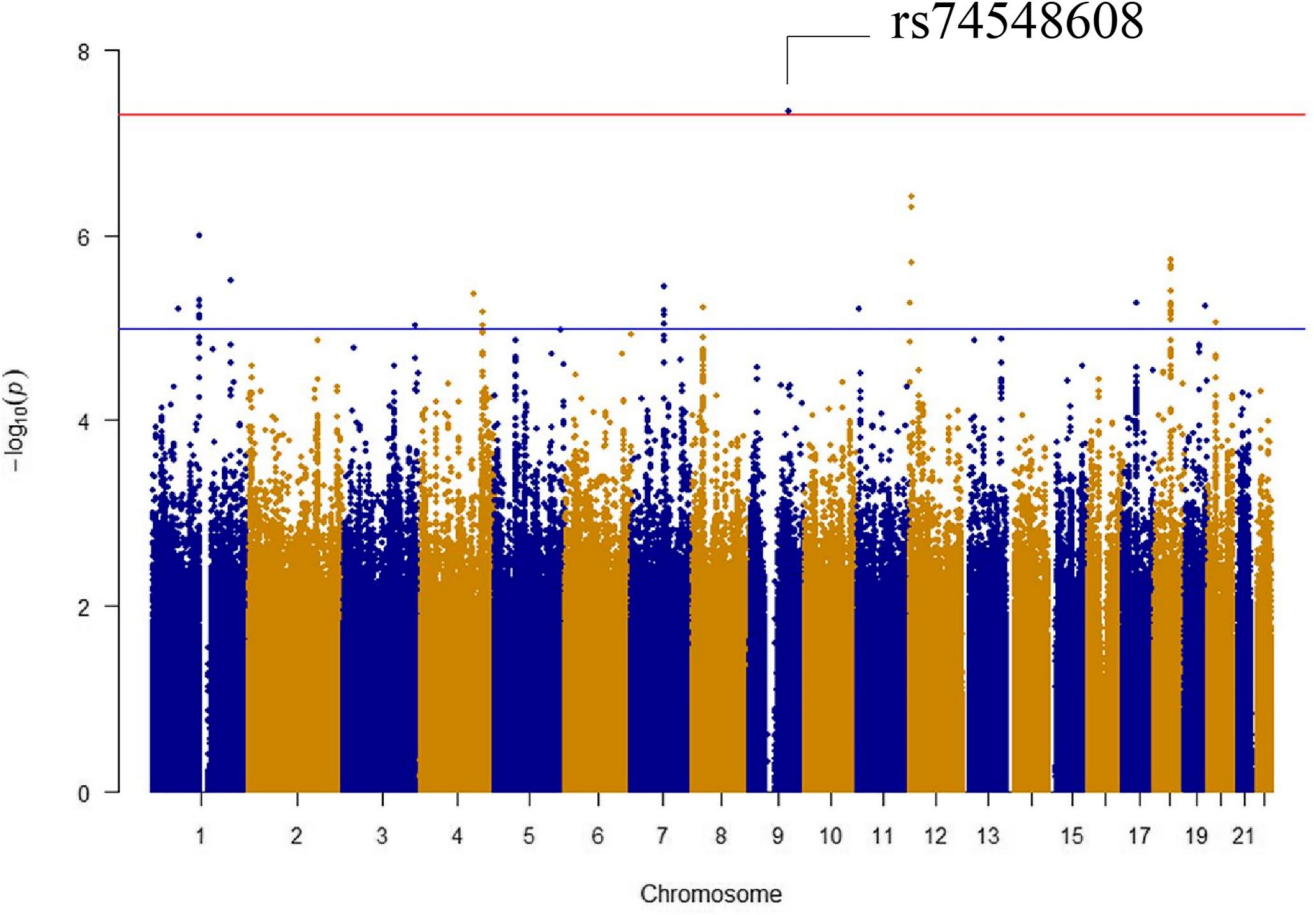
Manhattan plots of the association study in elderly group (Female older than 50). The red horizontal line indicates the genome‐wide significance threshold of *p* = 5 × 10^−8^ and the blue line indicates suggestive significance threshold of *p* = 1 × 10^−5^. The top SNP of genome‐wide significance threshold are marked by arrows with the SNP names

**FIGURE 2:**
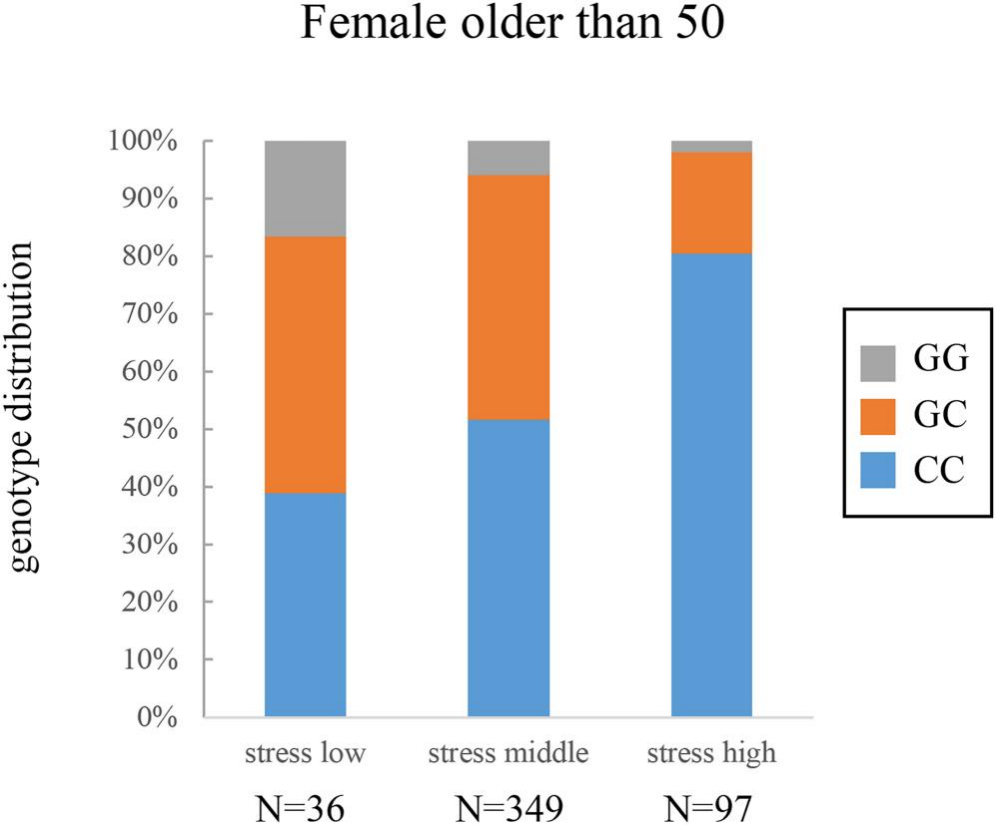
Distribution of the genotypes of the SNP. For each grade of perceived stress, the distribution of the rs74548608 genotypes is represented as vertical bars

Next, we conducted a GWAS using 3 datasets to investigate genetic factors involved in perceived stress of an individual. Concretely, all subjects (*n* = 1,040, mean age: 49.0 years) were adopted as the 1st dataset, the young group (558 aged < 50 years, mean age: 40.1 years) as the 2nd dataset, and the elderly group (482 aged ≥50 years, mean age: 59.3 years) as the 3rd dataset. As a result, no SNP meeting a genome‐wide significance level (*p* < 5 × 10^−8^) was detected on analyses involving all subjects or the young group, but an SNP exceeding the significance level (rs74548608) was detected on a GWAS involving the elderly group (Figure 1, Table 3). Furthermore, we analysed the distribution of rs74548608 genotypes in the elderly group, and confirmed that people with the C allele of SNP rs28392847 have a more stress compared with the G allele (Figure 2).

**TABLE 3:** Association results of perceived stress of an individual with rs74548608

Imputed or direct typing	SNP	Chr	Position	Ref	Alt	EA	Study subject	EAF	*β*	SE	*p*
Imputed	rs74548608	9	98406362	C	G	G	Whole female (*n* = 1040)	25.6	−0.104	0.0261	7.45E−05
							Female under 50 (*n* = 558)	26.3	−0.025	0.0365	0.4973
							Female older than 50 (*n* = 482)	24.8	−0.205	0.0369	4.584E−08

Abbreviations: Alt, alternative allele; *β*, beta coefficient; Chr, chromosome; EA, effect allele; EAF, effect allele frequency; *p*, *p*‐value; Ref, reference allele; SE, standard error.

**FIGURE 3:**
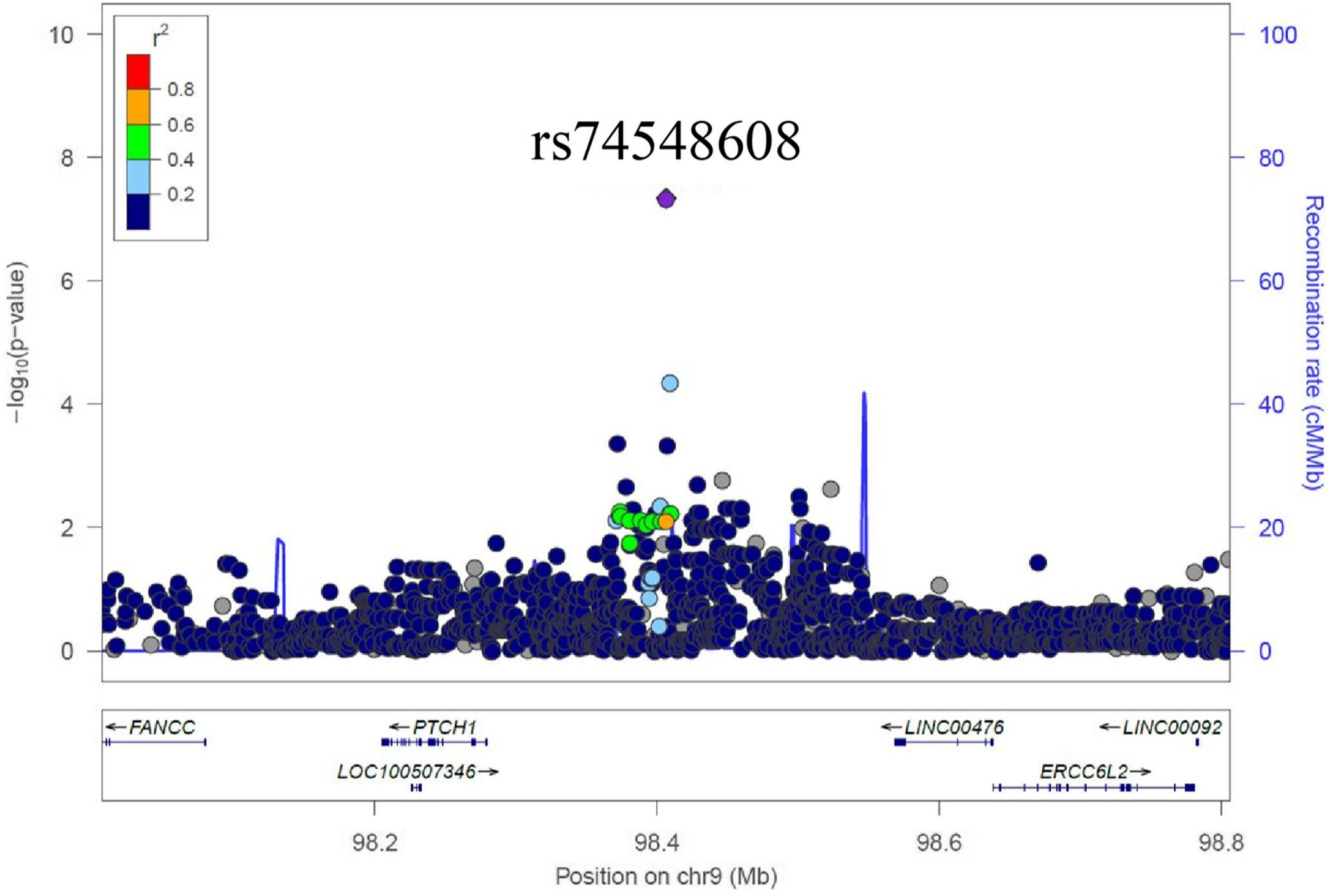
Regional association plot for rs74548608. Association results of both genotyped and imputed single nucleotide polymorphisms are plotted as the distribution of ‐log10 (*p*‐values) along the physical position on each chromosome. Results are shown in the region flanking 400 kb on either side of the marker SNP. rs74548608 is shown in purple, and the different colours indicate r2 values. The light blue line represents the recombination rate expressed in centiMorgan (cM) by Megabase (Mb). Genes in the region are represented at the bottom

Next, we prepared a Locus Zoom plot for an rs74548608‐adjacent area (Figure 3). In the rs74548608‐adjacent area, Patched‐1 (PTCH1), which is a gene that functions as a sonic hedgehog (Shh) receptor, and LINC00476, as a long non‐coding RNA, were present. On the other hand, when checking expression Quantitative Trait. Locus (eQTL) using the GTEx database (www.gtexportal.org/home/) and Haplo Reg 4.1 (https://pubs.broadinstitute.org/mammals/haploreg/haploreg.php), no gene of which the expression changes with rs74548608 was found.

## 5 DISCUSSION

In this study, we found rs74548608 as a SNP involved in perceived stress of an individual through a GWAS involving Japanese females (Figures 1, 2, 3, Table 3). This SNP is located on the upstream of PTCH1. No study has reported its function. No relevant gene was found on eQTL analysis. On the other hand, the signal transmission route of Shh, which acts as the ligand of PTCH1, regulates various cell processes.^22^ The expression of Shh in the brain tissue or nerve cells has been confirmed. Several studies indicated that Shh played an important role in nerve cell activity.^23^, ^24^, ^25^, ^26^ Furthermore, many studies suggested an association between Shh signalling and depression.^27^, ^28^, ^29^, ^30^ Although rs74548608 may influence the signal transmission route of Shh via PTCH1, the details must be further examined. On an analysis involving the subjects comprising the elderly group, rs74548608 met the genome‐wide significance level, but not on analyses involving all subjects or those comprising the young group. Such differences may have resulted from differences in the living environment and stress types between the young and elderly groups. The young group undergo a variety of events that the elderly group do not confront such as finding employment, marriage, and child rearing. In addition, a survey by the Ministry of Health, Labour and Welfare of Japan reported that stress related to work and education of children occupy a large proportion until the age of 50 years but decreases thereafter with increases in stress related to diseases and elderly care.^21^ Other factors, such as changes in the hormone balance associated with menopause, may also have affected the stress pattern. The average age of menopause in Japanese women is about 50 years,^31^ after which secretion of female hormones is known to markedly decrease, inducing various mental and physical changes. It has also been previously reported that the results of GWAS may differ in datasets from different age groups. For example, a previous GWAS regarding skin ageing with Chinese samples also showed that SNPs meeting the genome‐wide significance level differed between a dataset from all subjects (31–86 years, *n* = 1534) and that from subjects aged ≥50 years (50–86 years, *n* = 1084).^32^

Although rs74548608 is a candidate factor in the research of genetic factors of individual perceived stress, the results of the present study must be carefully interpreted until they are reproduced in other cohorts, because the number of samples was insufficient, and because the significance level was fulfiled only in the elderly group. In addition, perceived stress is considered likely to be a polygenic trait controlled by many genes, and the present study may have failed to detect multiple genes that only confer minor risks because of the small sample size. For the future, clarification of the entire picture of genetic factors involved in perceived stress by studies with more samples is awaited.

A past study carried out in the United States reported that the daily perceived stress is high in younger generations and decreases rapidly after the age of 50 years.^18^ A study in a Japanese population also showed that the percentage of people who feel stress decreases after the age of 50 years^21^ as it did in our study (Table 1). It is interesting that age‐associated changes in perceived stress were similar in countries with different racial and living environments. We hope that knowledge about stress and ageing deepens in the future by reproduction of our results in many countries and regions.

A limitation of this study was that it was based on responses to a self‐administered questionnaire rather than objective data collected by physicians. A self‐administered questionnaire survey may fail to detect many environmental factors that affect perceived stress. Analysis of more objective phenotype data obtained by physicians' examinations or a questionnaire with a battery of questions is considered necessary to clarify stress in future studies. Also, while the results of this study suggested that stress is related to skin ageing as in previous studies,^4^, ^5^ it cannot be denied that some subjects tended to give poor scores to both the stress level and skin quality. Also, while the present study was carried out in healthy subjects without serious diseases, we did not give attention to mild skin disorders or the use of drugs. The results of the present study must be interpreted carefully until they are confirmed by objective data such as measurements by dermatologists.

As observed above, we evaluated the effects of stress on the skin and searched for SNPs related to daily perceived stress in Japanese women. The SNP found in this study may be a candidate factor important for understanding the stress of Japanese. More detailed analysis should be promoted by increasing the sample size, involving the data obtained in this study.

## CONFLICT OF INTEREST

The authors declare no conflicts of interest associated with this manuscript.

## ETHICS STATEMENT

Prior to this study, its protocol was approved by the Ethics Review Board of our company.

## AUTHOR CONTRIBUTION

Yu Inoue: Conceptualization, Data curation, Formal analysis, Investigation, Writing – original draft. Yuichi Hasebe: Conceptualization, Project administration, Supervision, Writing – review & editing. Toshio Igarashi: Formal analysis, Investigation, Methodology, Software. Mika Kawagishi‐Hotta: Data curation, Formal analysis, Investigation, Methodology. Ryosuke Okuno: Data curation, Formal analysis, Investigation. Takaaki Yamada: Data curation, Methodology. Seiji Hasegawa: Conceptualization, Project administration, Supervision, Writing – review & editing.

## Supporting information

Figure S1

Table S1

## Data Availability

Author elects to not share data.
