# Supplementary figures and images for: Analysis of the effect of daily stress on the skin and search for genetic loci involved in the perceived stress of an individual
Source: Skin Health Dis. 2022 Apr 1;2(3):e110. doi: 10.1002/ski2.110 (PMC9435448; doi:10.1002/ski2.110)

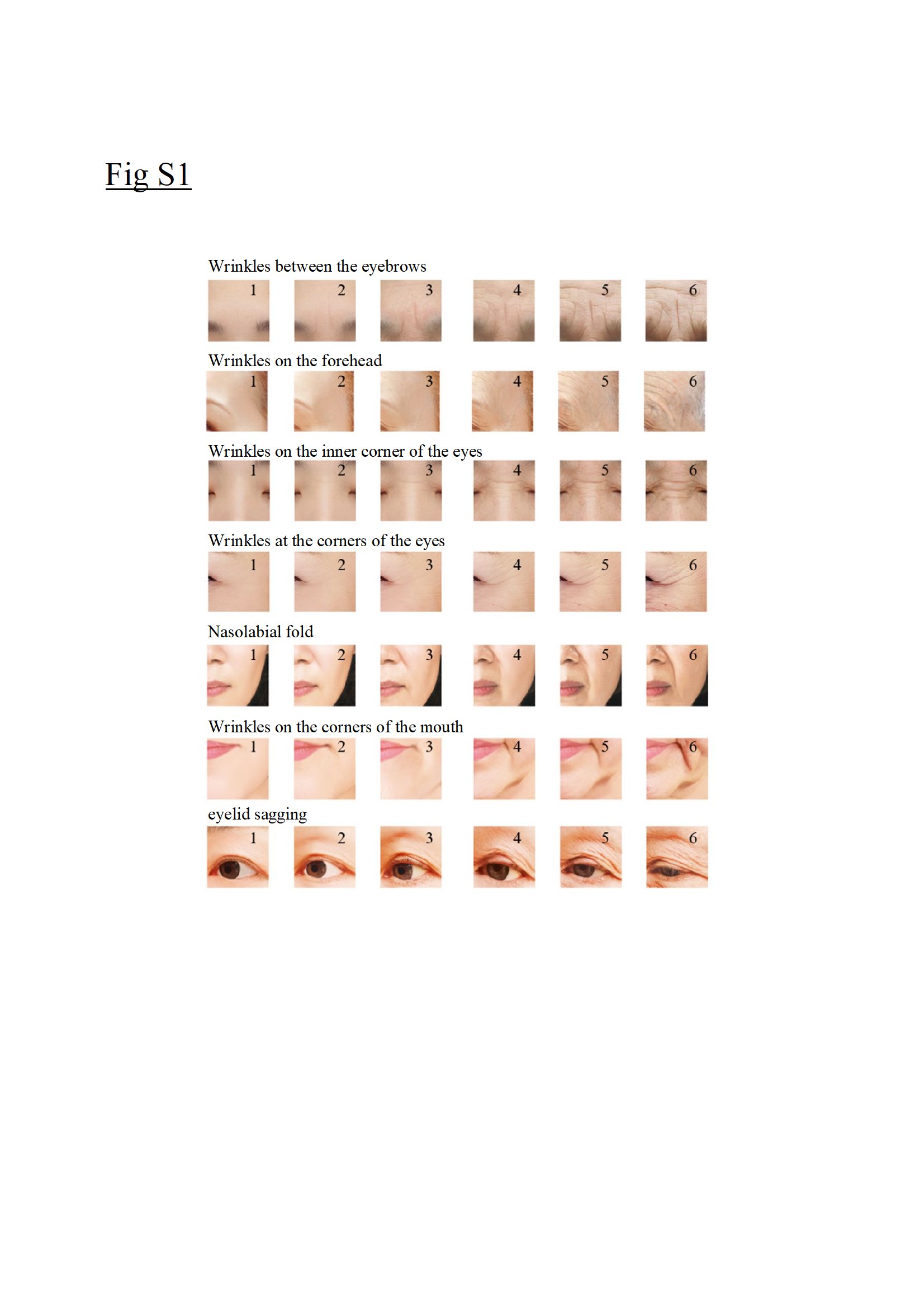

Supplement: Supplementary file 1 — Figure S1 [file SKI2-2-e110-s002.tif]
