# Supplementary material for: Analysis of the effect of daily stress on the skin and search for genetic loci involved in the perceived stress of an individual
Source: Skin Health Dis. 2022 Apr 1;2(3):e110. doi: 10.1002/ski2.110 (PMC9435448; doi:10.1002/ski2.110)
Supplement: Supplementary file 2 — Table S1 [file SKI2-2-e110-s001.docx]

| Skin conditon and  signs of skin aging | adjustments | | | | | |
| --- | --- | --- | --- | --- | --- | --- |
|  | age + sunscreen | | age + smoking | | age + sunscreen+ smoking | |
|  | β | *p* | β | *p* | β | *p* |
| Skin condition | 0.172 | 0.00000954 | 0.166 | 0.0000173 | 0.168 | 0.0000132 |
| Wrinkles between the eyebrows | 0.111 | 0.00527 | 0.110 | 0.00559 | 0.111 | 0.00500 |
| Wrinkles on the forehead | 0.0465 | 0.276 | 0.0479 | 0.261 | 0.0493 | 0.248 |
| Wrinkles on the inner corner of the eye | 0.0584 | 0.166 | 0.0578 | 0.171 | 0.0592 | 0.160 |
| Wrinkles at the corners of the eyes | 0.1057 | 0.0372 | 0.107 | 0.0345 | 0.108 | 0.0335 |
| Nasolabial fold | 0.1041 | 0.0450 | 0.102 | 0.0494 | 0.104 | 0.0455 |
| Wrinkles on the corners of the mouth | 0.0975 | 0.0429 | 0.0936 | 0.0527 | 0.0965 | 0.0452 |
| eyelid sagging | 0.0386 | 0.296 | 0.0375 | 0.310 | 0.0381 | 0.302 |

Table S1. Association results of the stress with skin condition and skin aging.

Association tests were adjusted for sunscreen and smoking habit in addition to age.

Abbreviations β, beta coefficient; *p, p*-value.
